# Comparison of the Effects of Early Versus Delayed Cord Clamping on Haemoglobin Levels in Neonates Delivered at Term

**DOI:** 10.7759/cureus.68714

**Published:** 2024-09-05

**Authors:** Fouzia Saghir, Sana Nazeef, Marina Khalid, Aisha Iqbal

**Affiliations:** 1 Obstetrics and Gynaecology, Aziz Bhatti Shaheed Teaching Hospital, Gujrat, PAK; 2 Obstetrics and Gynaecology, Services Institute of Medical Sciences, Lahore, PAK

**Keywords:** delayed cord clamping, delivered, early, hemoglobin, neonates, term

## Abstract

Background: The foetus is connected to the placenta via the umbilical cord, which enters through the abdomen at the umbilicus. A venous catheter for infusion and medication may be inserted via the umbilical vein, as it is directly related to the central circulation. Since delayed cord clamping increases iron storage after birth, it may help prevent anaemia in later infancy.

Methods: This randomized controlled trial was conducted in the Department of Obstetrics and Gynaecology at Aziz Bhatti Shaheed Teaching Hospital, Pakistan, over a period of six months, from August 2021 to January 2022. A total of 70 females were enrolled. The neonates were randomly divided into two groups using the lottery method. In group 1, neonates underwent early cord clamping, while in group 2, neonates underwent delayed cord clamping after delivery. Haemoglobin levels were noted and analysed using IBM SPSS Statistics for Windows, Version 20 (Released 2011; IBM Corp., Armonk, New York).

Results: The mean age of patients in the early cord clamping group was 26.80±7.59 years, and in the delayed cord clamping group, it was 28.14±6.12 years. The mean haemoglobin level in the early cord clamping group was 13.84±1.74, while in the delayed cord clamping group, it was 16.34±1.90 (p-value=0.001).

Conclusion: In neonates born at term, the mean haemoglobin level in the delayed cord clamping group was significantly higher than that in the early cord clamping group.

## Introduction

Half or more of children in underdeveloped nations have anaemia by the time they are 12 months old. In Pakistan, iron deficiency anaemia is one of the leading causes of infant mortality and morbidity. Since babies have more iron in their bodies at birth, delaying cord clamping helps prevent anaemia in later infancy [1,2]. Delayed cord clamping is a controversial procedure used globally during the third stage of labour management. Such trial studies are highly necessary in Pakistan due to the lack of national guidelines and sufficient data on the subject [3]. It appears that delaying cord clamping after delivery is safe and will likely reduce the rate of anaemia among newborns in our community [4]. Compared to early umbilical cord clamping, delaying cord clamping after delivery may improve infant health [5].

Policies regarding the timing of cord clamping vary; some recommend it within the first 60 seconds after delivery, while others suggest waiting more than one minute after birth or until cord pulsation has stopped before clamping the umbilical cord [6,7]. There is ongoing debate about whether the cord should be clamped early or late in the third stage of labour. Maternal and neonatal factors, as well as gestational age, are important considerations when deciding the timing of umbilical cord clamping. Evidence suggests that delaying cord clamping can increase newborn blood volume by 30% at delivery, thereby enhancing placental transfusion. This may reduce the incidence of anaemia in term infants by increasing iron storage, but it may also increase the likelihood of phototherapy and jaundice [8]. Delaying cord clamping may reduce the risks of intraventricular haemorrhage and late-onset sepsis, as well as decrease the need and severity of blood transfusions to treat anaemia in premature infants. Another potential benefit of delayed cord clamping is a reduction in alloimmunization in Rh-negative women; however, this has not been thoroughly tested or validated [9].

One randomized controlled trial reported that in early cord clamping, the mean haemoglobin level was 175±19 g/L, while in delayed cord clamping, the mean haemoglobin level was 189±17 g/L, with the difference being statistically significant (P<0.05) [10]. Another study reported a mean haemoglobin level of 16.97±1.134 g/L in early cord clamping and 19.59±1.395 g/L in delayed cord clamping, also finding a significant difference (P<0.05) [11]. However, one trial found no significant difference in mean haemoglobin levels between the early (10.6±1.1 g/dL) and three-minute delay groups (10.7±1.0 g/dL), with a P-value greater than 0.05 [12].

This research aims to examine the mean haemoglobin levels in term neonates delivered by early versus delayed cord clamping. Although the current literature presents contradictory findings, it has generally been observed that delayed cord clamping results in higher haemoglobin levels compared to early cord clamping. By conducting this research, we aim to resolve the debate and verify that delayed cord clamping is indeed beneficial. Our goal is to enhance current practices, collect updated evidence, and eventually incorporate delayed cord clamping into standard practice to ensure better health outcomes for neonates.

## Materials and methods

This randomized controlled trial was conducted in the Department of Obstetrics and Gynaecology at Aziz Bhatti Shaheed Hospital, Gujrat, Pakistan, over six months, from August 2021 to January 2022.

A sample size of 70 cases, with 35 cases in each group, was calculated with a 95% confidence interval, 80% power of the test, and taking the expected magnitude of the mean haemoglobin level, i.e., 175±19 g/L with early cord clamping and 189±17 g/L with delayed cord clamping in neonates delivered at term [11]. The sampling technique used was non-probability, consecutive sampling.

Sample selection

Inclusion Criteria

Neonates delivered at term (gestational age >37 weeks) to females aged 18-40 years with a parity of less than 5, presenting with a singleton pregnancy, were included in the study.

Exclusion Criteria

Females with chronic or gestational hypertension, preeclampsia, eclampsia, gestational diabetes, or a previous caesarean section; anaemic females; those with maternal infection; or those indicated for emergency caesarean section, i.e., pathological cardiotocography at the time of admission.

Data Collection Procedure

After obtaining approval from the hospital ethical committee, 70 females fulfilling the selection criteria were enrolled in the study from the labour room. Informed consent was obtained. Demographic information (name, age, gestational age, parity, and contact) was recorded. The neonates were then randomly divided into two groups using the lottery method. Cord clamping was defined as early if the cord was clamped within 10 seconds after birth and as delayed if the cord was clamped after three minutes of birth. In group 1, neonates underwent early cord clamping, while in group 2, neonates underwent delayed cord clamping after delivery. The neonates were followed up in the post-natal wards for 24 hours. After 24 hours, a blood sample from the neonate was drawn with the help of a 3 cc syringe under aseptic measures. All samples were sent to the hospital laboratory for assessment of haemoglobin levels. Reports were assessed, and haemoglobin levels were noted. All this information was collected through a pre-designed proforma.

Data Analysis

Data were analysed using IBM SPSS Statistics for Windows, Version 20 (Released 2011; IBM Corp., Armonk, New York). Both groups were compared using an independent sample t-test for mean haemoglobin levels. A p-value ≤0.05 was considered significant. Data were presented in the form of tables.

## Results

The mean age of the early cord clamping patients was 26.80±7.59 years, while in the delayed cord clamping patients, it was 28.14±6.12 years. In our study, the mean gestational age of the early cord clamping patients was 38.94±0.84 weeks, and in the delayed cord clamping patients, it was 38.97±0.71 weeks. According to this study, the mean BMI in the early cord clamping group was 21.51±3.93 kg/m², and in the delayed cord clamping group, it was 23.92±3.74 kg/m². In the early cord clamping group, there were 13 (37.1%) primigravida, 13 (37.1%) with parity 1-2, and 9 (25.7%) with parity 3-4. In the delayed cord clamping group, there were 8 (22.9%) primigravida, 22 (62.9%) with parity 1-2, and 5 (14.3%) with parity 3-4 (Table 1).

**Table 1:** Basic information of females in both groups

Demographics	Study Groups	
	Early cord clamping	Delayed cord clamping
n	35	35
Age (years)	26.80 ± 7.59	28.14 ± 6.12
Gestational age (weeks)	38.94 ± 0.84	38.97 ± 0.71
BMI (kg/m^2^)	21.51 ± 3.93	23.92 ± 3.74
Parity		
Primigravida	13 (37.1%)	8 (22.9%)
Parity 1-2	13 (37.1%)	22 (62.9%)
Parity 3-4	9 (25.7%)	5 (14.3%)

In this study, the mean haemoglobin level in the early cord clamping group was 13.84±1.74 g/L, while in the delayed cord clamping group, it was 16.34±1.90 g/L. A statistically significant difference was found between the study groups in haemoglobin levels (p-value = 0.001) (Table 2).

**Table 2:** Comparison of haemoglobin in both groups

Variables	Study Groups		p-value
	Early cord clamping	Delayed cord clamping	
n	35	35	
Haemoglobin (g/l)	13.84 ± 1.74	16.34 ± 1.90	0.001

The study results showed that in patients aged ≤30 years, the mean haemoglobin level in the early cord clamping group was 13.92±1.97 g/L, while in the delayed cord clamping group, it was 15.78±1.94 g/L. Similarly, in patients aged >30 years, the mean haemoglobin level in the early cord clamping group was 13.705±1.33 g/L, while in the delayed cord clamping group, it was 17.540±1.16 g/L. A statistically significant difference was found between the study groups in haemoglobin levels stratified by age (p-value <0.05). The study results also showed that in patients with a gestational age of 38 weeks, the mean haemoglobin level in the early cord clamping group was 13.70±2.01 g/L, while in the delayed cord clamping group, it was 16.17±1.71 g/L. In patients with a gestational age of 39 weeks, the mean haemoglobin level in the early cord clamping group was 14.30±1.51 g/L, while in the delayed cord clamping group, it was 15.98±1.96 g/L. Similarly, in patients with a gestational age of 40 weeks, the mean haemoglobin level in the early cord clamping group was 13.55±1.69 g/L, while in the delayed cord clamping group, it was 17.32±1.85 g/L. A statistically significant difference was found between the study groups in haemoglobin levels stratified by gestational age (p-value <0.05). In patients with primary parity, the mean haemoglobin level in the early cord clamping group was 13.682±1.75 g/L, while in the delayed cord clamping group, it was 15.201±1.69 g/L. Similarly, in patients with multi-parity, the mean haemoglobin level in the early cord clamping group was 14.01±1.77 g/L, while in the delayed cord clamping group, it was 17.54±1.28 g/L. A statistically significant difference was found between the study groups in haemoglobin levels stratified by parity (p-value <0.05). In patients with normal BMI, the mean haemoglobin level in the early cord clamping group was 14.21±1.73 g/L, while in the delayed cord clamping group, it was 16.54±2.45 g/L. Similarly, in patients with abnormal BMI, the mean haemoglobin level in the early cord clamping group was 13.50±1.73 g/L, while in the delayed cord clamping group, it was 16.092±0.92 g/L. A statistically significant difference was found between the study groups in haemoglobin levels stratified by BMI (p-value <0.05) (Table 3).

**Table 3:** Comparison of haemoglobin levels in both groups stratified by effect modifiers

Variables	Categories	Study groups		p-value
		Early cord clamping	Delayed cord clamping	
Age (years)	≤30	13.92 ± 1.97	15.78 ± 1.94	0.002
	>30	13.705 ± 1.33	17.540 ± 1.16	0.001
Gestational age (weeks)	38	13.70 ± 2.01	16.17 ± 1.71	0.007
	39	14.30 ± 1.51	15.98 ± 1.96	0.022
	40	13.55 ± 1.69	17.32 ± 1.85	0.001
Parity	Primary	13.682 ± 1.75	15.201 ± 1.69	0.012
	Multiple	14.01 ± 1.77	17.54 ± 1.28	0.001
BMI	Normal	14.21 ± 1.73	16.54 ± 2.45	0.003
	Abnormal	13.50 ± 1.73	16.092 ± 0.92	0.001

## Discussion

In the current randomised controlled experiment, the mean haemoglobin level with early vs delayed cord clamping in term newborns was compared at Unit II, Department of Obstetrics & Gynaecology, Aziz Bhatti Shaheed Hospital, Gujrat. During the third stage of labour, the placenta is fully expelled from the body after the baby is born. If bone marrow transplantation is not an option, umbilical cord blood is a valuable source of highly proliferative stem cells, such as haematopoietic stem cells. Despite the limited value of blood banking for future autologous or familial use, the public is advised to save cord blood. Theoretically, the arterial and venous blood gases of the umbilical cord might be affected by delaying cord clamping [13].

In this study, the mean haemoglobin level in the early cord clamping group was 13.84±1.74 g/L, and in the delayed cord clamping group, it was 16.34±1.90 g/L, which is statistically significant with a p-value of 0.001. Delaying cord clamping is likely to improve newborn outcomes for both term and preterm children, according to research by Garofalo and Abenhaim. This holds true even in regions where neonatal iron deficiency anaemia is uncommon. Nonetheless, at this time, there is not enough data to recommend delaying cord clamping in newborns who are not vigorous but still need resuscitation [13].

One randomised controlled trial reported that in early cord clamping, the mean haemoglobin level was 175±19 g/L, while in delayed cord clamping, the mean haemoglobin level was 189±17 g/L. The difference was reported to be significant (P<0.05) [10]. Compared to early cord clamping, which exposes children to more red blood cell transfusions, delayed cord clamping in babies with severe CHD seems to be both safe and practical, according to research by Backes. Neither the gestational age at delivery nor the time of operation varied between the groups that had early or delayed cord clamping [5]. In another study, it was reported that in early cord clamping, the mean haemoglobin level was 16.97±1.134 g/L, while in delayed cord clamping, the mean haemoglobin level was 19.59±1.395 g/L. The difference was reported to be significant (P<0.05) [11].

Researchers McDonald and Middleton found no evidence that the risk of postpartum haemorrhage increases with a two- to three-minute delay in cord clamping. Furthermore, newborns in areas with limited access to nutritious food may benefit from delayed cord clamping, as it improves their iron status; however, there is an increased risk of jaundice requiring phototherapy if clamping is delayed [14]. Even in regions where cases of newborn iron deficiency anaemia are uncommon, research conducted by Garofalo and Abenhaim showed that delaying cord clamping is likely to improve neonatal outcomes for term and preterm children [13]. Nonetheless, at this time, there is not enough data to recommend delaying cord clamping in newborns who are not vigorous but still need resuscitation.

McDonald et al. also found that there should be more leeway in delaying umbilical cord clamping in healthy full-term infants. This is especially true given the mounting evidence that this practice boosts infants' early haemoglobin concentrations and iron stores. Assuming phototherapy for jaundice is readily accessible, delaying cord clamping is probably a good idea [15]. Additionally, research conducted by the American College of Obstetricians and Gynecologists found no link between delayed umbilical cord clamping and any of the following outcomes: increased risk of postpartum haemorrhage, increased blood loss during delivery, different levels of haemoglobin after delivery, or the necessity of blood transfusions [16].

Another trial reported no significant differences in mean haemoglobin values, 10.6±1.1 g/dL and 10.7±1.0 g/dL, between the groups with early cord clamping and those with a three-minute delay, respectively (P>0.05) [12].

During the trial, we also observed a few limitations, including a small sample size and a limited number of outcome variables, such as haemoglobin. However, multiple outcomes could have been observed during the study. Due to time and financial constraints, we could not include other variables, such as the impact of cord clamping timing on haematocrit levels, any change in the body temperature of the neonate, or later development of infections or cerebrovascular changes.

## Conclusions

Based on this research, the average haemoglobin level was considerably greater in neonates born at term who had delayed cord clamping compared to those who underwent early cord clamping. In the future, we will advocate for delayed cord clamping as a means to enhance the well-being of newborns. Further trials should be conducted in different centres, and multicentric data should be generated to confirm the beneficial aspects of the timing of cord clamping.
